# Predictive Role of Venous Drainage and Lesion Margins in Bronchopulmonary Sequestration Classification

**DOI:** 10.3390/jcm14093018

**Published:** 2025-04-27

**Authors:** Giada Pedroni, Giulia Albo, Francesca Galbiati, Irene Maria Borzani, Andrea Zanini, Ernesto Leva, Francesco Macchini, Stefano Mazzoleni

**Affiliations:** 1 Pediatric Surgery, ASST Grande Ospedale Metropolitano Niguarda, 20162 Milan, Italy; giada.pedroni@ospedaleniguarda.it (G.P.); giulia.albo@ospedaleniguarda.it (G.A.); francesco.macchini@ospedaleniguarda.it (F.M.); stefano.mazzoleni@ospedaleniguarda.it (S.M.); 2 Pediatric Surgery, Fondazione IRCCS Ca’ Granda Ospedale Maggiore Policlinico, 20122 Milan, Italy; francesca.galbiati1@unimi.it (F.G.); ernesto.leva@policlinico.mi.it (E.L.); 3 Pediatric Radiology, Fondazione IRCCS Ca’ Granda Ospedale Maggiore Policlinico, 20122 Milan, Italy; irene.borzani@policlinico.mi.it

**Keywords:** congenital lung malformation, bronchopulmonary sequestration, thoracoscopy, pediatric surgery

## Abstract

**Background**: Bronchopulmonary sequestration (BPS) is a portion of dysplastic pulmonary tissue not communicating with the tracheobronchial tree. Its vascularization is provided by abnormal arteries originating from the systemic circulation. Previous papers report different venous drainage (VD) between intralobar (ILS) and extralobar sequestration (ELS), respectively, into the pulmonary or the systemic veins. The aim of our study is to investigate the VD as independent predictor of the type of PS. **Methods**: We retrospectively reviewed 41 pediatric patients who underwent surgery for BPS between 2016 and 2023 in two centers. Patients treated prenatally and without pre-operative CT were excluded. BPS were classified either intra or extra lobar. Pre-operative radiological BPS classification prediction was assessed based on intra-operative thoracoscopic findings. Lesion VD and sharp margins (SM) on pre-operative CT were assessed as predicting factors. **Results**: A total of 36 patients were included: 24 ILS and 12 ELS. All patients underwent thoracoscopic resection without major complications. VD is significantly different between ILS and ELS: 96% of ILS drain into the pulmonary system while 83% of ELS drain into a systemic vein (*p* < 0.00001). On pre-operative CT, the absence of SM predicts for an ILS in 100% of cases. SM has a PPV for ELS of 85.7%. The combination of SM and systemic VD has a PPV for ELS of 91.7%. **Conclusions**: In our series, the absence of SM alone is suggestive for an ILS in 100% of cases, while the combination of SM and systemic VD is more accurate in predicting ELS. This may help improving surgical planning and family consultation.

## 1. Introduction

Bronchopulmonary sequestration (BPS) is a rare lung congenital anomaly that represents from 0.15% to 6.4% of all congenital pulmonary malformation [1,2]. This condition is characterized by a segment or lobe of dysplastic lung tissue separated from the rest of tracheobronchial tree that receives an abnormal blood supply, typically derived from systemic vessels rather than the pulmonary circulation.

A description of a similar condition dates back to 1861 by Rokytansky and Rektorik; however, it was not thoroughly described until 1946 by Pryce et al. [3,4]. Subsequent advancements in radiological imaging and thoracic surgery have greatly enhanced our understanding of this pathology. BPS can be categorized into two main forms as intralobar sequestration (ILS) and extralobar sequestration (ELS), depending on their location in relation to the normal lung parenchyma.

ILS is the more common form of BPS, with a reported incidence of 75% to 86% [5]; this type is located within a normal lung tissue with which it shares the visceral pleura. On the other hand, the ELS has its own visceral pleura, maintaining complete anatomic separation from adjacent normal lung, often located in abnormal areas such as mediastinum, diaphragm or, rarely, the abdomen.

BPS exhibits a wide spectrum of presentations, ranging from asymptomatic incidental finding to variable degree of symptoms, depending on the size and location of the lesion. Younger patients, especially neonates and children, are often more affected, with pulmonary sequestration being diagnosed incidentally during an evaluation for respiratory symptoms [6]. ILS is often associated with recurrent infections or respiratory difficulties due to the poor functionality of the sequestrated tissue [7,8,9]. In adults, pulmonary sequestration may remain latent for years before becoming symptomatic [10].

The standard treatment involves surgical resection of the lesion, through sequestrectomy in cases of ELS or lobectomy or atypical resection for ILS [11].

Therefore, based on the significant difference in surgical techniques between the two forms of BPS, an accurate preoperative evaluation is essential for proper surgical planning. For this reason, it would be essential to study what may be the distinctive aspects of ILS compared to ELS on preparatory imaging. In particular, in the recent literature, the venous component of the aberrant vascularization of BPS has not been as widely investigated as the arterial component. The original description of BPS venous drainage was reported by Stocker et al., 1986 [12]: this study states that the venous drainage of intralobar sequestrations is mainly into the pulmonary vein, while extralobar sequestrations most commonly have systemic venous drainage. These findings have been widely cited in the literature and have provided a foundational framework for differentiating between these two forms of sequestration based on vascular characteristics. Despite numerous references to the study of Stocker et al., these findings have not been subsequently verified nor investigated as pre-operative predicting factors. Currently, no method can surely preoperatively distinguish ILS from ELS.

Therefore, the primary aim of our study is to investigate venous drainage as independent predictor of the type of BPS. Secondary aims are to investigate for other potential predictive findings on pre-operative CT scan and to verify Stocker’s original description of different venous drainage between ILS and ELS.

Our study intends to provide new evidence that could contribute to more accurate pre-operative diagnosis and optimized therapeutic management for patients affected by this rare pulmonary condition.

## 2. Materials and Methods

We conducted a retrospective multicenter study. We reviewed clinical data of all pediatric patients with pulmonary sequestration who underwent surgical treatment between January 2012 and December 2023 in two Centers: Grande Ospedale Metropolitano Niguarda and Policlinico Fondazione Ca’ Granda in Milan, Italy.

According to our protocol, the patients with CLM that are identified during morphological ultrasound (US) are followed up with an US. In cases of hydrothorax or fetal hydrops, a fetal MRI is performed between the 21st and 30th weeks of gestation. After birth, in asymptomatic patients, a chest X-ray is performed on day 1 of life. In the case of normal radiological findings, after an ECHO and cardiology assessment, the newborn is discharged and a thoracic MRI is planned within the first month of life using the “feed&wrap” method. In this way, we can investigate the lesion and exclude potential differential diagnosis while maintaining a safe profile with no radiation and no sedation required.

Patients with positive MRI are scheduled for a preoperative contrast-enhanced chest CT scan between third and fourth months of life, which usually requires a brief sedation.

Thoracoscopic resection is performed around between the fourth and sixth month of life. After discharge, patients are followed up in the out-patient clinic at 1 week, 1 month, and then every 3 months in the first year of life and subsequently every 6 months in the second year of life. A single chest X-ray is routinely performed one month after surgery to verify complete lung re-expansion and to exclude early postoperative complications. Subsequent follow-up evaluations are primarily clinical and conducted by a multidisciplinary team. These visits aim to monitor respiratory function, growth, and overall development, ensuring long-term outcomes are closely supervised.

Outborn patients referred to our center undergo the “feed&wrap” MRI if they are referred before 6 weeks of age. Those patients referred afterwards undergo pre-operative CT scan and surgical resection as described [13].

The inclusion criteria for this study were as follows pediatric patients diagnosed with pulmonary sequestration who underwent surgical intervention, the availability of preoperative computed tomography imaging and complete clinical and surgical records.

Patients who had undergone prenatal treatment with fetal laser therapy and those with insufficient or unavailable preoperative radiological imaging data were excluded from this study.

### 2.1. Imaging and Radiological Evaluation

Preoperative CT imaging with contrast enhancement were reviewed. Imaging studies were conducted using multi-detector CT scanners, ensuring high-resolution visualization of pulmonary structures and vascular anatomy. Parameters such as slice thickness, contrast medium dosage, and timing were standardized to optimize visualization of the sequestrated tissue and associated vascular supply.

All CT scans were blinded reviewed by a single expert radiologist, who was not aware of the final intraoperative finding. This strategy was adopted to ensure consistency in the interpretation of radiological features.

The parameters evaluated on CT scans included the pattern of venous drainage (systemic or pulmonary); the origin of the anomalous artery supplying the lesion; the presence or absence of sharp margin [14] (a radiological sign that refers to the clear and well-defined boundaries around the lesion, making it easily distinguishable from the surrounding tissues); the component of the lesions (cystic air or solid) and the presence of pulmonary hyperinflation.

All patients underwent preoperative CT imaging using a shared, standardized protocol across both institutions. The protocol included contrast-enhanced, high-resolution chest CT scans with consistent acquisition parameters, allowing optimal visualization of vascular structures and lesion characteristics. Moreover, all surgical procedures were performed by the same pediatric surgeon operating at both centers, ensuring uniformity in the surgical approach and intraoperative assessment. This consistency in both radiological and surgical practice contributed to the reliability and homogeneity of the data collected.

### 2.2. Data Collection and Classification

The following parameters was systematically collected from the medical records:

- Demographic parameters: age and sex;
- Diagnosis: the type of pulmonary sequestration (ILS or ELS) was noted based on final surgical findings and histopathological confirmation;
- The type of venous drain rated on preoperative CT and the type found intraoperatively.

### 2.3. Statistical Analysis

We used Fisher’s test to assess the statistical significance of correlations between categorical variables, such as the type of venous drainage (pulmonary vs. systemic), the presence or absence of sharp margins, and the composition of the lesions (e.g., the presence of a cystic air component) versus the type of sequestration (ILS or ELS).

Additionally, the *p*-value was calculated to determine the statistical significance of the correlations, using a significance threshold set at less than 0.01.

To assess the diagnostic reliability of radiological features in predicting the type of BPS, the positive predictive value (PPV) and negative predictive value (NPV) were calculated. These values allowed us to measure how accurately the characteristics observed in the preoperative CT predict the presence of a certain BPS subtype. In particular, PPV and NPV were calculated for systemic venous drainage, presence of sharp margins and their combination in diagnosis of ELS.

Finally, a radiological concordance analysis was performed, comparing the diagnoses made by the radiologist based on the preoperative CT images with the intraoperative and histopathological findings.

## 3. Results

In the study period, a total of 41 pediatric patients underwent surgical treatment for BPS.

All patients underwent surgery using a thoracoscopic approach. No cases required conversion to open thoracotomy. Furthermore, no postoperative complications were observed in our cohort.

Of all these patients, five were excluded from the study as they did not meet the inclusion criteria. Two patients had undergone prenatal treatment with fetal laser therapy. Preoperative documentation was unavailable for three patients: two of them had initially been treated for congenital diaphragmatic hernia (CDH), and BPS was identified as an incidental finding during surgery without prior preoperative CT study; the third patient had been referred from another region, and the preoperative CT scan was not available for this retrospective consultation.

The study included a total of 36 patients: 24 females (66.7%). The mean age at surgery was 6.0 months. In these cases, the definitive diagnosis of the BPS subtype, determined by the concordance between CT parameters, intraoperative findings, and histopathological confirmation, identified 67% (24/36) of patients as ILS and 33% (12/36) as ELS.

Initially, we analyzed the intraoperative findings and the features on the preoperative CT scans independently; subsequently, we combined every feature found on the CT scans with the diagnosis of ILS or ELS and for each we looked for statical significance with Fisher’s test. Finally, we combined the results.

Regarding the intraoperative findings, venous drainage into the pulmonary vein was observed in 25 cases, while into the systemic circulation in 11 cases.

Preoperative CT scans identified a systemic arterial supply in all 36 cases (3 cases originating from the celiac trunk, 1 case from abdominal aorta and 32 cases from thoracic aorta). Regarding the margins of the lesion, 22 cases exhibited the absence of sharp margins, whereas well-defined margins were observed in 14 lesions. Analysis of the composition of the BPS revealed a cystic air component in 7 cases. Additionally, hyperinflation of the surrounding normal pulmonary tissue was documented in 18 cases.

Analyzing the correlation between venous drainage observed on preoperative CT scans and the definitive diagnosis of the BPS subtype, we found that of the patients diagnosed with ILS, 96% (23/24) were found to have venous drainage into the pulmonary vein, while 4% (1/24) had drainage into the systemic circulation. Regarding the patients diagnosed with ELS, 83% (10/12) presented with venous drainage into the systemic circulation and 17% (2/12) into the pulmonary veins. This correlation was analyzed using Fisher’s test, demonstrating statistical significance, with a *p*-value < 0.00001. The presence of systemic venous drainage for identifying an ELS had a PPV of 90.9% and an NPV of 92%.

In the same way, we analyzed the correlation between diagnosis of ILS or ELS with the presence or absence of sharp margin viewed on CT scans: of the 14 lesions with well-defined margins, 86% (12/14) were ELS and 14% (2/14) as ILS; on the other hand, of the 22 lesions without sharp margins, 100% (22/22) were ILS. A Fisher’s test for this variable showed a statistical significatively with *p*-value < 0.00001; the presence of sharp margins had a positive predictive value (PPV) for identifying an ELS of 85.7% and an NPV of 100%.

Regarding the composition of the lesions on CT scans, of the seven lesions with cystic air composition observed, 100% (7/7) were ILS. Analyzing that correlation with Fisher’s test we did not find a statical significance.

Combining the systemic venous drainage and the presence of sharp margins we found that the PPV for diagnosing ELS increased to 91.7% while the NPV to 95.8%.

Finally, the radiologist who conducted a blind review of the preoperative CT scans of all the patients in the study accurately described 23 out of the 24 ILS lesions; however, 1 lesion was misjudged as an ELS. Similarly, among the 12 lesions diagnosed as ELS, 11 were correctly identified by the radiologist as ELS, and 1 lesion was incorrectly classified as ILS. The radiologist’s diagnostic accuracy based only on preoperative CT scan was 94.4%.

Raw data can be found as Supplementary Material.

## 4. Discussion

BPS is a rare and complex congenital anomaly of the lung, accounting for a small percentage of all congenital pulmonary malformations. It is traditionally classified into ILS and ELS.

Accurate preoperative classification of BPS subtypes remains elusive, yet the radiological features enabling an accurate preoperative diagnosis have been insufficiently explored in the scientific literature.

The diagnosis of BPS relies on a combination of imaging modalities [15]. Chest radiography is typically the first diagnostic step, useful for suspecting the presence of a lung lesion and excluding other major anomalies. On X-ray, pulmonary sequestration appears as a well-defined area of consolidation in the lung. However, an X-ray alone is insufficient to determine the exact nature and location of the sequestration. High-resolution computed tomography (HRCT) is one of the most important diagnostic tools in the suspicion of BPS, providing detailed visualization of lung morphology, showing the lesion as a homogeneous or heterogeneous mass separated from normal lung tissue and enabling the identification of the lesion’s margins. It also allows for the assessment of the lesion composition and detection of hyperinflation in adjacent lung tissue, which may indicate emphysematous changes probably due to air trapping in the “transition zone” between the lesion and normal lung tissue [15]. Moreover, CT plays an important role in evaluating the abnormal vascularization of the BPS, which is essential for surgical planning. The success of surgical management largely depends on the preoperative identification and intraoperative control of the aberrant vascular supply. Aberrant arteries must be carefully ligated and divided to prevent intraoperative hemorrhage; since vascular variations are common, preoperative CT analysis is crucial for anticipating and managing intraoperative challenges.

Magnetic resonance imaging (MRI), though less commonly used than CT, can be valuable in selected cases. MRI provides detailed lung structure visualization without ionizing radiation, but MRI, in particularly in pediatric patients requires sedation. In our institution, MRI is used in the neonatal period [16] without sedation, taking advantage of the newborn’s spontaneous sleep, to perform a differential diagnosis to exclude other conditions that may mimic BPS, such as pulmonary vascular malformations, benign lung tumors, and chronic infections. However, CT scan remains the main diagnostic method for studying BPS in the preoperative setting.

The first suspicion of BPS in the prenatal period is raised with US and is then further explored with fetal MRI [17,18,19].

A clear preoperative classification would enable more precise therapeutic decisions, because the therapeutic management of BPS is primarily dictated by the type of sequestration (ILS or ELS).

ELSs, being lesions separated from the lung parenchyma, are suitable for sequestrectomy without the need for lobar resection. On the other hand, for ILS, due to the intimate integration of sequestrated tissue with adjacent normal lung parenchyma, surgical management often requires lobectomy or atypical resection. We therefore underline the importance of a preoperative distinction between the two subtypes of BPS, for better surgical planning and for a more precise parental counseling.

Complete resection of the sequestrated tissue is essential in order to reduce the complications of BPS, in particular the recurrent infections for ILS or acute hemothorax and malignant degeneration for ELS, two rare complications described in the literature [20,21].

Both techniques can be approached through thoracotomy or via video-assisted thoracoscopic procedures (VATS) [22,23]. In our center, a minimally invasive approach is always the first choice, and all patients in our study underwent a thoracoscopic procedure. VATS reduces postoperative pain, length of hospital stay and recovery times [24,25].

In recent years, endovascular embolization and coiling of aberrant systemic arteries have emerged as viable non-invasive alternatives to surgical resection [26,27]. Embolization involves the occlusion of aberrant arteries using coils, plugs, or liquid embolic agents, effectively cutting off the blood supply to the sequestrated tissue. However, this procedure is associated with its own complications, including femoral arterial access site thrombosis and transient limb ischemia, non-target embolization of the pulmonary arteries and aorta [28], post-embolization syndrome with fever and pain [29]. Another technique in the treatment of BPS is atypical resection, which aims to be a conservative technique that involves the removal of the lesion without removing healthy lung tissue. The distinction between normal and abnormal tissue to be resected is not easy to determine, but it could be facilitated by the use of indocyanine green (ICG) [30,31]. This technique may also offer potential advantages in terms of accuracy and safety. However, the long-term efficacy in preventing recurrent infections and other complications associated with BPS is still under investigation.

In the treatment of BPS, the use of fetal laser can also be considered. Although most fetuses with BPS have a prenatal course free from adverse events, a small percentage may incur into potentially fatal complications, mainly hydrothorax, with an overall mortality rate of 68%. Intrauterine ultrasound-guided laser coagulation (USLC) has been considered a valid treatment for fetuses who have presented this complication [32,33]. In our cohort, two patients were excluded from the study as they had been prenatally treated with fetal laser therapy.

Through our retrospective analysis of 36 pediatric patients, we have highlighted key radiological markers that can aid in preoperative diagnosis and ultimately assist in the surgical management of BPS. The characteristics of venous drainage, the presence or absence of sharp margins and the identification of cystic components within the lesion were evaluated as key diagnostic features to differentiate between ILS and ELS. These parameters were systematically analyzed to determine their independent and combined diagnostic value, providing critical insights into the morphological and anatomical distinctions that underpin these two subtypes of pulmonary sequestrations.

### 4.1. Venous Drainage as a Diagnostic Marker

**Figure 1:**
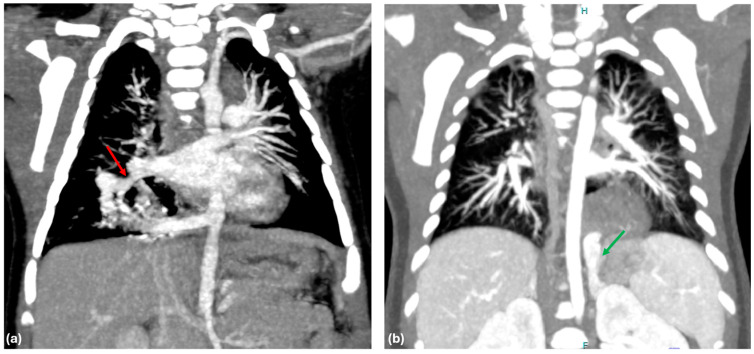
(**a**) CT scan showing a right lower ILS draining in to right inferior pulmonary vein (red arrow: pulmonary venous drainage); (**b**) CT scan showing a left lower ELS draining into the left adrenal vein (green arrow: systemic venous drainage).

One of the main findings of our study is the significant difference in venous drainage between ILS and ELS, which offers substantial evidence for the importance of venous drainage patterns in predicting the type of BPS. Our results show that 96% of ILS cases drain into the pulmonary vein (Figure 1a), whereas 83% of ELS cases have systemic venous drainage (Figure 1b). This observation supports the hypothesis proposed by Stocker et al. in 1986 [12]. Interestingly, it is important to note that a small percentage of patients did not conform to the expected drainage patterns, for instance, 4% of ILS patients exhibited systemic venous drainage, while 17% of ELS patients demonstrated pulmonary venous drainage. These outliers underscore the complexity of BPS and suggest that venous drainage alone may not provide a definitive classification in all cases.

These exceptions may be attributed to embryological variations in the development of pulmonary and systemic venous systems [34]. Additionally, the venous outflow may follow the path of least resistance, influenced by the lesion’s anatomical position relative to nearby vessels [35]. Moreover, mixed or borderline forms of sequestration—cases that blur the classical ILS/ELS distinction—have been well described. Lastly, imaging limitations, especially in complex or subtle cases, may contribute to misinterpretation of venous drainage [36].

### 4.2. Sharp Margins as a Radiological Indicator

**Figure 2:**
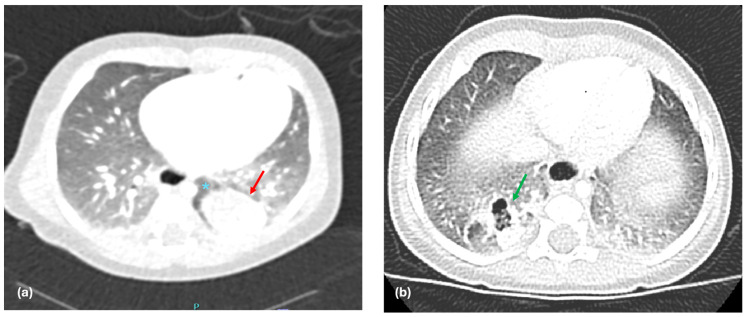
(**a**) CT scan showing a left lower ELS with sharp margins (red arrow: sharp margins of the lesion; blue asterisk: area of lung hyperinflation). (**b**) CT scan showing a right lower ILS with undefined margins (green arrow: undefined margins of the lesion).

In general, sharp margins could be suggestive of a lesion that is enclosed by its own visceral covering or another anatomic structure, thereby separating it from the surrounding parenchyma. This feature has been widely studied in various medical contexts, where sharp margins often imply benign or well-contained growths [37,38]. However, its specific relevance and application to the diagnosis of BPS have been less extensively explored in the literature. In the context of ELS, the presence of sharp margins may reflect the separation from the normal lung due to the presence of its own pleural covering. Given this characteristic, sharp margins in BPS may suggest the presence of an ELS (Figure 2a), where the abnormal lung tissue is fully encased in its own pleural membrane, as opposed to ILS, where the sequestrated tissue is embedded within the normal lung tissue without such an enclosure (Figure 2b). In our study, we chose to include the analysis of sharp margins as a radiological parameter to help differentiate between the two subtypes of PS. In our study, the presence of sharp margins was shown to have a strong correlation with the presence of ELS, with a positive predictive value (PPV) of 85.7% and NPV of 100%. This suggests that sharp margins are a key feature in distinguishing ELS from ILS on preoperative imaging.

### 4.3. Combined Radiological Features for Improved Preoperative Diagnosis

**Figure 3:**
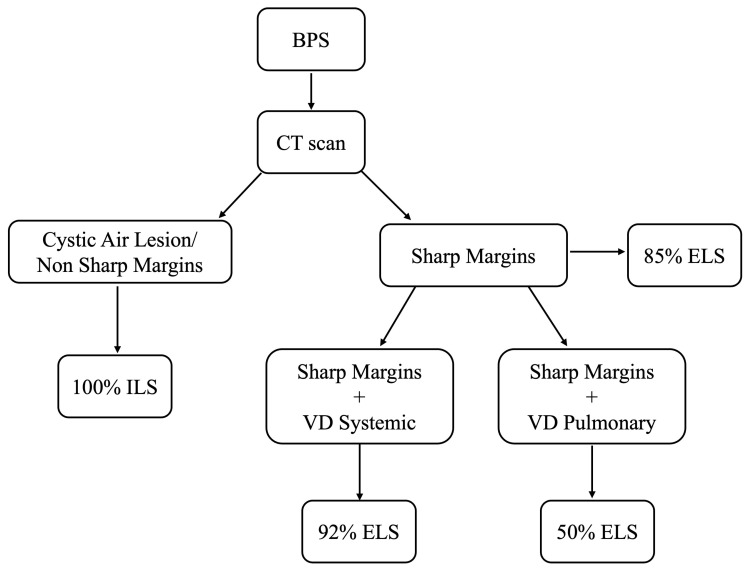
Diagnostic algorithm with predictive value.

Combining the different features of the preoperative CT, we have seen, as outlined in our flowchart (Figure 3), that, when the margins of the lesion were well defined, the PPV for ELS is 85%, but when we combined these parameters with systemic venous drainage, the PPV for identifying ELS increased to 91.7%. Finally, if the lesions did not show sharp margins or presented cystic aerial composition, the PPV for ILS was 100%. This demonstrates the importance of an integrated radiological assessment, which considers various parameters to reach a more precise diagnosis and further supports the use of specific features of CT in the preoperative planning of BPS.

Although even if not predictive on a statistical level, cystic composition may still be relevant in guiding the management strategy. Previous studies have reported that cystic features can influence both diagnostic suspicion and surgical decision-making, particularly when distinguishing between BPS and other congenital lung malformations such as CPAM, bronchogenic cysts, or post-infective pneumatocele [39,40,41]. In fact, the presence of air-filled cysts may lead to a more extensive surgical approach due to the higher risk of infection or diagnostic uncertainty.

### 4.4. Radiologist Accuracy in Preoperative Differentiation of BPS Subtypes

Finally, we asked the expert radiologist to diagnose the type of BPS based on the preoperative CT alone, without being informed of the definitive diagnosis. The radiologist accurately described 23 out of the 24 ILS lesions, with only 1 lesion misjudged as an ELS. This misjudgment likely stemmed from an atypical presentation of venous drainage (in this case, the drainage occurred in the systemic circulation) and from the sharp margins showed by the lesion. Similarly, among the 12 lesions diagnosed as ELS, 11 were correctly identified by the radiologist as ELS, and 1 lesion was incorrectly classified as ILS. This misclassification may have occurred due to pulmonary venous drainage of the lesion. The radiologist’s accuracy, with a success rate of 94.4%, highlights the reliability of CT as a support tool in the preoperative classification of BPS.

However, misclassifications suggest that there are still challenges to be addressed. Although CT is a valid diagnostic tool, its interpretation can be complex and requires careful analysis, especially in cases with unconventional radiological features.

### 4.5. Clinical and Surgical Implications

The preoperative identification of BPS type—whether ILS or ELS—has significant implications for surgical management. Understanding, preoperatively, the anatomical characteristics of the BPS, can provide surgeons with valuable information when planning the surgical approach. Our study indicates that preoperative CT imaging, when used in conjunction with the patterns of venous drainage and sharp margins, can help predict the type of sequestration and inform the choice of surgical technique. For instance, the combination of systemic venous drainage and sharp margins on CT is highly suggestive of ELS, which may require a much less complex surgical approach than ILS. By using these radiological features as predictive markers, surgeons can better prepare for the challenges presented by each case, potentially reducing intraoperative complications, improving patient outcomes and presented a better information to parents.

While surgical planning represents a direct application of our findings, their clinical value extends well beyond the operating room. A more accurate preoperative classification between ILS and ELS improves parental counseling by allowing a clearer explanation of the expected procedure, associated risks, and recovery timeline. Additionally, it reduces intraoperative uncertainty and supports more informed surgical decision-making. Importantly, in some institutions where conservative or delayed strategies—such as endovascular embolization or clinical observation—are considered, a reliable radiological distinction may significantly influence patient management. These broader implications highlight the relevance of our model not only in the surgical setting but also in optimizing comprehensive, patient-centered care.

### 4.6. Limitations and Future Directions

While our study provides valuable insights into the role of venous drainage and sharp margins in the classification of BPS, there are several limitations to consider. Firstly, our study is retrospective in nature, and the data were collected from two specific centers, which may limit the generalizability of the findings. A multicenter prospective study would provide more robust evidence to support our conclusions and may help validate the predictive value of venous drainage and sharp margins in other populations.

Secondly, the sample we analyzed is relatively small, due to the rarity of the condition. This limitation highlights the need for further research. Conducting a larger, prospective study, as already stated above, would be highly beneficial, as it would provide more robust data and help validate the findings and concepts presented in our current study.

Another limitation of our study is that the CT images were reviewed by a single radiologist. While this ensured internal consistency and minimized inter-observer variability, it does not account for potential differences in interpretation. Future prospective studies should include multi-reader analyses and assess inter-rater reliability to validate the reproducibility of the radiological predictors across different levels of expertise.

Lastly, although we found a strong association between venous drainage and BPS classification, there remains a degree of overlap between the two types of sequestration. As mentioned earlier, some patients with ILS exhibited systemic venous drainage, and some patients with ELS had pulmonary venous drainage. This variability highlights the complexity of PS and suggests that additional factors may also play a role in determining the classification and clinical outcomes of PS.

Further research on new radiological and clinical markers is promising and is needed to explore additional radiological features that may enhance the accuracy of preoperative diagnosis, which could lead to significant advancements in surgical plannings.

## 5. Conclusions

Our study demonstrates that venous drainage and sharp margins are valuable radiological features in the classification of BPS. The significant differences in venous drainage between ILS and ELS, along with the diagnostic value of sharp margins, provide meaningful evidence for their use in preoperative imaging. By combining these radiological features, clinicians can more accurately predict the type of sequestration. This may help improving the surgical planning and family consultation.

## Data Availability

The dataset supporting the conclusions of this article are available as Supplementary Materials.

## Supplementary Materials

The following supporting information can be downloaded at: https://www.mdpi.com/article/10.3390/jcm14093018/s1.

## Abbreviations

The following abbreviations are used in this manuscript:

BPS	Broncho-pulmonary sequestration
ELS	Extra-lobar sequestration
ILS	Intra-lobar sequestration
VATS	Video-assisted thoracoscopy

## References

[1] Gabelloni M., Faggioni L., Accogli S., Aringhieri G., Neri E. (2021). Pulmonary sequestration: What the radiologist should know. Clin. Imaging.

[2] Pederiva F., Rothenberg S.S., Hall N., Ijsselstijn H., Wong K.K.Y., von der Thüsen J., Ciet P., Achiron R., Pio d’Adamo A., Schnater J.M. (2023). Congenital lung malformations. Nat. Rev. Dis. Primers.

[3] Chakraborty R.K., Modi P., Sharma S. (2025). Pulmonary Sequestration. StatPearls.

[4] Pryce D.M. (1946). Lower accessory pulmonary artery with intralobar sequestration of lung; a report of seven cases. J. Pathol. Bacteriol..

[5] Sarkar S., Girija A., Shirgaonkar R., Mohapatra P.R. (2024). Intralobar pulmonary sequestration. QJM Mon. J. Assoc. Physicians.

[6] Jaiswal L.S., Neupane D. (2021). Pulmonary sequestration presenting as a massive haemoptysis in adult: A case report. Int. J. Surg. Case Rep..

[7] Coutinho D.J., Dias M.C., Oliveira M.J., Vaz D.C., Shiang M.T. (2015). Bronchopulmonary sequestration presenting as a spontaneous pneumothorax. Respir. Care.

[8] Aryal G., Pathak V. (2011). Bronchopulmonary sequestration presenting as recurrent pneumonia. WMJ.

[9] Hussain K., Suleman S., Fatimi S.H., Khan J.A. (2024). Bronchopulmonary sequestration presenting as haemoptysis with an aberrant blood supply from the pulmonary artery. BMJ Case Rep..

[10] Naffaa L., Tank J., Ali S., Ong C. (2014). Bronchopulmonary sequestration in a 60 year old man. J. Radiol. Case Rep..

[11] Hegde B.N., Tsao K., Hirose S. (2022). Management of Congenital Lung Malformations. Clin. Perinatol..

[12] Stocker J.T. (1986). Sequestrations of the lung. Semin. Diagn. Pathol..

[13] Macchini F., Borzani I., Cavalli S., Morandi A., D’Angelo I.D., Zanini A., Ferrari C., Ichino M., Leva E. (2020). Thoracoscopic Resection of Congenital Lung Malformation: Looking for the Right Preoperative Assessment. Eur. J. Pediatr. Surg. Off. J. Austrian Assoc. Pediatr. Surg. Z. Kinderchir..

[14] Xu J., Napel S., Greenspan H., Beaulieu C.F., Agrawal N., Rubin D. (2012). Quantifying the margin sharpness of lesions on radiological images for content-based image retrieval. Med. Phys..

[15] Mughal A.Z., El-Zeki A., Habib A.M. (2024). The role of 3-dimensional reconstruction imaging in bronchopulmonary sequestration. Multimed. Man. Cardio-Thorac. Surg..

[16] Wani S.A., Mufti G.N., Bhat N.A., Baba A.A. (2015). Pulmonary Sequestration: Early Diagnosis and Management. Case Rep. Pediatr..

[17] Cho M.K., Lee M.Y., Kang J., Kim J., Won H.S., Lee P.R., Jeong E., Lee B.S., Kim E.A., Yoon H. (2020). Prenatal sonographic markers of the outcome in fetuses with bronchopulmonary sequestration. J. Clin. Ultrasound.

[18] Ghobrial P.M., Levy R.A., O’Connor S.C. (2011). The fetal magnetic resonance imaging experience in a large community medical center. J. Clin. Imaging Sci..

[19] Epelman M., Kreiger P.A., Servaes S., Victoria T., Hellinger J.C. (2010). Current imaging of prenatally diagnosed congenital lung lesions. Semin. Ultrasound CT MRI.

[20] Preziosi A., Morandi A., Galbiati F., Scanagatta P., Chiaravalli S., Fagnani A.M., Di Cesare A., Macchini F., Leva E. (2022). Acute haemothorax and pleuropulmonary blastoma: Two extremely rare complications of extralobar pulmonary sequestration. J. Pediatr. Surg. Case Rep..

[21] Yarlagadda S., Mandava A., Fonseca D., Koppula V. (2024). Mucoepidermoid Carcinoma of the Lung in Intralobar Bronchopulmonary Sequestration. Radiol. Cardiothorac. Imaging.

[22] Macchini F., Zanini A., Morandi A., Ichino M., Leva E. (2020). Thoracoscopic Surgery for Congenital Lung Malformation Using Miniaturized 3-mm Vessel Sealing and 5-mm Stapling Devices: Single-Center Experience. J. Laparoendosc. Adv. Surg. Tech. Videoscop..

[23] Wang D., Mou Y., Wang J. (2022). Comparing open and thoracoscopy approach for the treatment of pulmonary sequestration in children. Pediatr. Surg. Int..

[24] Macchini F. (2020). Thoracoscopic resection of congenital pulmonary airway malformations: Timing and technical aspects. J. Thorac. Dis..

[25] Kiblawi R., Zoeller C., Zanini A., Ure B.M., Dingemann J., Kuebler J.F., Schukfeh N. (2021). Video-Assisted Thoracoscopic or Conventional Thoracic Surgery in Infants and Children: Current Evidence. Eur. J. Pediatr. Surg. Off. J. Austrian Assoc. Pediatr. Surg. Al Z. Kinderchir..

[26] Zener R., Bottoni D., Zaleski A., Fortin D., Malthaner R.A., Inculet R.I., Mujoomdar A. (2017). Transarterial embolization of intralobar pulmonary sequestration in a young adult with hemoptysis. J. Thorac. Dis..

[27] Borzelli A., Paladini A., Giurazza F., Tecame S., Giordano F., Cavaglià E., Amodio F., Corvino F., Beomonte Zobel D., Frauenfelder G. (2017). Successful endovascular embolization of an intralobar pulmonary sequestration. Radiol. Case Rep..

[28] Ganeshan A., Freedman J., Hoey E.T.D., Steyn R., Henderson J., Crowe P.M. (2010). Transcatheter coil embolisation: A novel definitive treatment option for intralobar pulmonary sequestration. Heart Lung Circ..

[29] Lee B.S., Kim J.T., Kim E.A.R., Kim K.S., Pi S.Y., Sung K.B., Yoon C.H., Goo H.W. (2008). Neonatal pulmonary sequestration: Clinical experience with transumbilical arterial embolization. Pediatr. Pulmonol..

[30] Motono N., Iwai S., Funasaki A., Sekimura A., Usuda K., Uramoto H. (2019). Indocyanine green fluorescence-guided thoracoscopic pulmonary resection for intralobar pulmonary sequestration: A case report. J. Med. Case Rep..

[31] Preziosi A., Paraboschi I., Giuliani S. (2023). Evaluating the Development Status of Fluorescence-Guided Surgery (FGS) in Pediatric Surgery Using the Idea, Development, Exploration, Assessment, and Long-Term Study (IDEAL) Framework. Child.

[32] Zanini A., Macchini F., Boito S., Morandi A., Ferrara G., Persico N., Leva E. (2022). Intrauterine Ultrasound-Guided Laser Coagulation as a First Step for Treatment of Prenatally Complicated Bronchopulmonary Sequestration: Our Experience and Literature Review. Eur. J. Pediatr. Surg. Off. J. Austrian Assoc. Pediatr. Surg. Al Z. Kinderchir..

[33] Tse W.T., Poon L.C., Wah Y.M., Hui A.S.Y., Ting Y.H., Leung T.Y. (2021). Bronchopulmonary sequestration successfully treated with prenatal radiofrequency ablation of feeding artery. Ultrasound Obstet. Gynecol..

[34] Langston C. (2003). New concepts in the pathology of congenital lung malformations. Semin. Pediatr. Surg..

[35] Lee E.Y., Boiselle P.M., Cleveland R.H. (2008). Multidetector CT evaluation of congenital lung anomalies. Radiology.

[36] Biyyam D.R., Chapman T., Ferguson M.R., Deutsch G., Dighe M.K. (2010). Congenital lung abnormalities: Embryologic features, prenatal diagnosis, and postnatal radiologic-pathologic correlation. Radiographics.

[37] Schaible J., Meiler S., Poschenrieder F., Scharf G., Zeman F., Rennert J., Pregler B., Knobloch C., Kleine H., Grote S. (2020). Sharp margin and geographic shape: Systematic evaluation of two novel CT features in COVID-19 pneumonia. BJR Open.

[38] Ilsen B., Vandenbroucke F., Beigelman-Aubry C., Brussaard C., de Mey J. (2016). Comparative Interpretation of CT and Standard Radiography of the Pleura. J. Belg. Soc. Radiol..

[39] Stocker J.T., Madewell J.E., Drake R.M. (1977). Congenital cystic adenomatoid malformation of the lung. Classification and morphologic spectrum. Hum. Pathol..

[40] Pogoriler J., Vargas S.O. (2025). Cystic masses of the pediatric lung: Update on congenital pulmonary airway malformation and its differential diagnosis. Virchows Arch..

[41] Vinayak T.P., Mohanty S., Das K. (2022). ‘Hybrid’ Bronchopulmonary Malformation—Lobar Emphysema and Extra Lobar Sequestration. Fetal Pediatr. Pathol..

